# Preoperative dexamethasone administration in hepatectomy of 25-min intermittent Pringle’s maneuver for hepatocellular carcinoma: protocol for a randomized controlled trial

**DOI:** 10.1186/s13063-023-07820-0

**Published:** 2023-11-30

**Authors:** Yang Huang, Liangliang Xu, Ning Wang, Xingyu Pu, Wentao Wang, Tianfu Wen, Mingqing Xu, Li Jiang

**Affiliations:** Division of Liver Surgery, Department of General Surgery, West China Hospital, Sichuan University, Sichuan Province, Chengdu, 610041 China

**Keywords:** Dexamethasone, Hepatocellular carcinoma, Ischemia-reperfusion injury, Hepatectomy, Perioperative outcome

## Abstract

**Background:**

Our previous randomized controlled trial (RCT) have demonstrated that intermittent Pringle’s maneuver (IPM) with a 25-min ischemic interval can be applied safely and efficiently in open or laparoscopic hepatectomy in patients with hepatocellular carcinoma (HCC) patients. But prolonging the hepatic inflow blocking time will inevitably aggravate the ischemia-reperfusion injury (IRI) caused by systemic response. This RCT aims to evaluate the effect of administration of dexamethasone versus placebo before clamping the hilar pedicle on postoperative liver function, inflammatory response, and perioperative outcomes among HCC patients undergoing liver resection with 25-min hepatic inflow occlusion.

**Methods and analysis:**

This will be a randomized, dual-arm, parallel-group, double-blinded trial. All eligible and consecutive patients are coming from a regional medical center who are diagnosed with HCC and underwent radical R0/R1 resection. All participates are randomly allocated in dexamethasone group or placebo group. All surgeons, anesthesiologists, and outcome assessors will be blinded to allocation status. Primary endpoints are transaminase-based postoperative hepatic injury on seven consecutive days after surgery and assessed by their peak values as well as area under the curve (AUC) of the postoperative course of aminotransferases. Secondary endpoints are postoperative total bilirubin (TBil), coagulation function, inflammatory cytokines and their respective peaks, intraoperative blood loss, postoperative hospital stay, morbidity, and mortality. The above parameters will be compared using the corresponding statistical approach. Subgroup analysis will be performed according to the liver cirrhosis and major hepatectomy.

**Discussion:**

Based on our previous study, we will explore further the effect of glucocorticoid administration on attenuating the surgical stress response in order to follow securely 25-min hepatic inflow occlusion. Therefore, the trial protocol is reasonable and the results of the trial may be clinically significant.

**Trial registration:**

This trial was registered on 3 December 2022, in the Chinese Clinical Trial Registry (http://www.chictr.org.cn), ChiCTR2200066381. The protocol version is V1.0 (20221104).

**Supplementary Information:**

The online version contains supplementary material available at 10.1186/s13063-023-07820-0.

## Background

At present, the control of intraoperative bleeding is still a crucial topic in hepatectomy. Specialists have tested various methods in attempts to decrease blood loss, transfusion requirements, and morbidity during hepatectomy [1]. The IPM is one of the most common methods of vascular control strategies which is a cycle of 15-min inflow occlusion followed by 5-min reperfusion [2, 3]. One major concern of applying the IPM is IRI to the remnant liver [4, 5]. Although 15-min inflow occlusion has been considered a standard time for IPM [6], the optimal occlusion and reperfusion time to balance blood loss and IRI is pending. There are four RCTs comparing the perioperative outcome with prolonged IPM time versus standard 15-min IPM time [7–10]. Maartje et al. [7], Esaki et al. [8], and Kim et al. [9] reported that 30-min IPM induced the hepatocellular injury, and inflammatory responses are similar to that in 15-min IPM in patients who underwent liver resection, but their studies are limited to the case selection or sample size. One of them is our research, which demonstrates that IRI caused by 25-min IPM is not inferior to the 15-min IPM with a large sample of HCC patients and 25-min IPM is related to lower blood loss and higher speed for parenchyma transection [10]. We choose 25 min as the ischemic interval, because in East Asia, most HCC patients are accompanied with varying degrees of cirrhosis and liver function impairment.

Glucocorticoids, such as methylprednisolone, hydrocortisone, and dexamethasone, are proved to be effective drugs that modulate level of inflammatory cytokines and attenuate IRI stress [11]. But there are currently no international guidelines recommending the routine use of glucocorticoids when clamping the hepatic hilum during hepatectomy. In a systematic review of steroids in liver resection published in 2014 [12], Richardson et al. reported five RCTs. There was a total of 379 patients, with 190 patients in the pre-operative steroid group and 189 in the placebo group. The sample size was 33, 53, 73, 20, and 200, respectively. More than half of the patients came from one study from Japan. All five studies referred to post-operative complications, length of stay, post-operative serum total bilirubin (TBil), and serum prothrombin time (PT). There was a statistically significant reduction in post-operative TBil associated with the use of steroids [*P* = 0.05, OR: − 0.43, 95% confidence interval (CI): − 1.04 to − 0.015]. Data were available in four of the studies pertaining to post-operative serum interleukin-6 (IL-6). There was a significant reduction in serum IL-6 in association with steroid administration (*P* = 0.01, OR: − 46.4, 95% CI: − 83.4 to − 9.39). What is more, in the past two decades, RCTs of preoperative administration of glucocorticoids in patients undergoing liver resection have demonstrated favorable postoperative changes in systemic inflammation, including interleukin-10 (IL-10), tumor necrosis factor-α (TNF-α), C reactive protein (CRP), and liver function parameters, such as aspartate amine transferase (AST), alanine amine transferase (ALT), and PT [13–18]. Moreover, perioperative glucocorticoids administration can decrease the incidence of postoperative complications [13, 17].

Although the above studies have demonstrated that preoperative glucocorticoids administration may improve perioperative outcome for patients undergoing liver resection, evidence supporting improved postoperative outcome of prolonging the ischemic interval time in HCC patients with cirrhosis is lacking. Therefore, we design the trial to evaluate the effect of dexamethasone versus placebo on postoperative short-term outcome in HCC patients undergoing liver resection with 25-min IPM. Meanwhile, we will explore the effect of dexamethasone on HCC patients with cirrhosis.

## Methods and analysis

### Study design

This is a single-center, parallel, double-blinded, randomized controlled trial. Liver function, inflammatory cytokines, and perioperative outcomes will be compared between two groups (intravenous 10 mg dexamethasone versus equivalent saline) before 10 min of hepatic inflow occlusion to evaluate the effect of dexamethasone in HCC patients undergoing liver resection with 25-min IPM. All participants will be blinded to the grouping. This study will be conducted in accordance with the principles of the Declaration of Helsinki and conform to the “Standard Protocol Items: Recommendations for Interventional Trials” checklist [19].

### Study setting

This trial will be conducted at West China Hospital of Sichuan University in Chengdu, Sichuan, China. West China Hospital of Sichuan University is one of the largest regional medical centers in China, which performs more than 2000 liver resections for HCC per year. The Department of Liver Surgery is national key specialty, which is staffed by seven well-trained liver surgeons with over 20-year experience in liver surgery, four of whom will participate in the study. The investigators in the trial are responsible for completing the operation, managing and interpreting of the data, and writing of the final report and have the decision to submit the report for publication and have ultimate authority over any of these activities.

### Recruitment

**Fig. 1:**
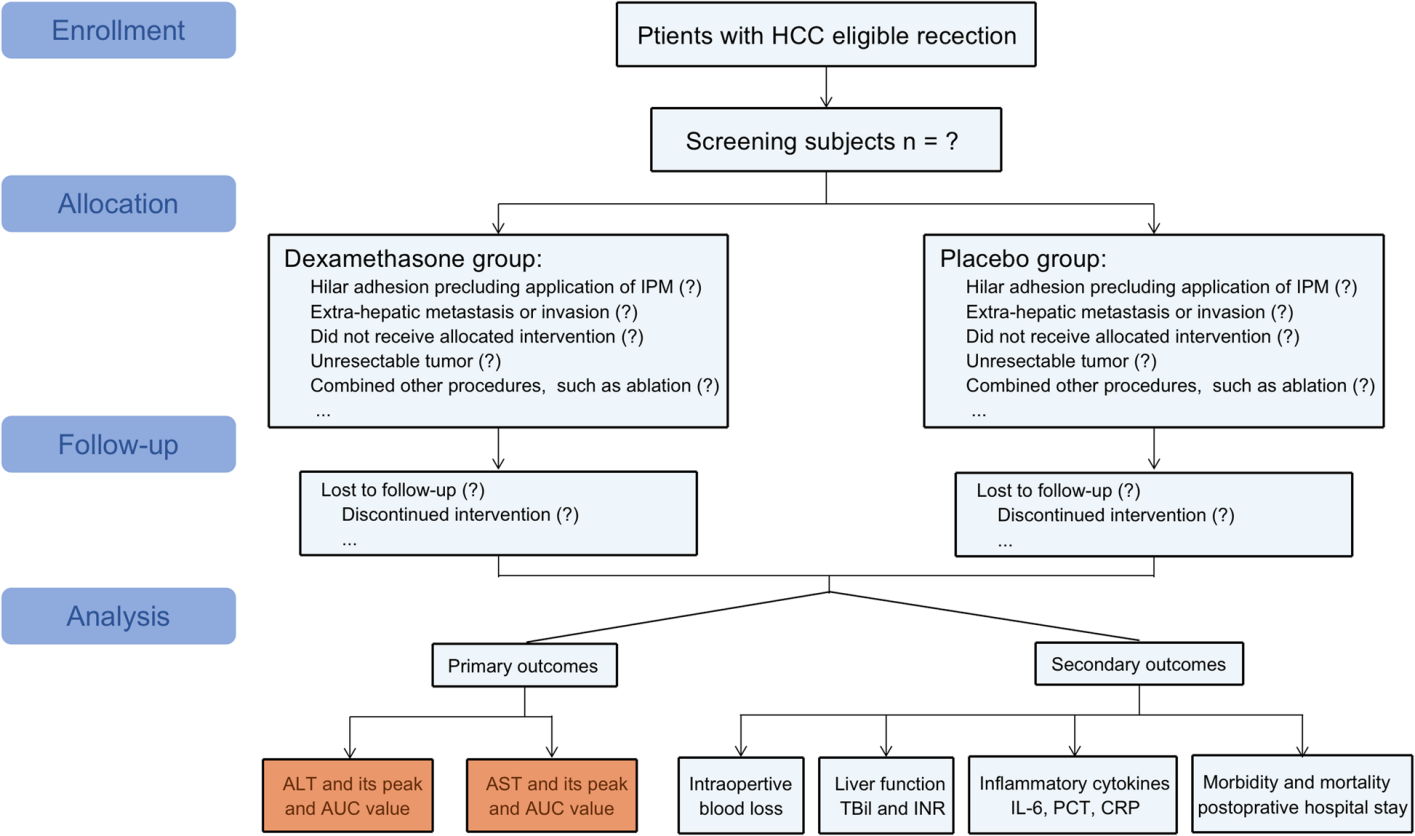
Flowchart of the study protocol. HCC, hepatocellular carcinoma; IPM, intermittent Pringle’s maneuver; ALT, alanine amine transferase; AUC, area under the curve; AST, aspartate amine transferase; TBil, total bilirubin; INR, international normalized ratio; IL-6, interleukin-6; PCT, procalcitonin; CRP, C reactive protein

This recruitment will be conducted from 3 December 2022 to 1 April 2023. A total of 270 consecutive patients who are eligible for the inclusion and exclusion criteria will be enrolled in this study. When physicians preliminarily evaluate the patient diagnosed with HCC at the outpatient clinic to be able to treat surgically, a trained research assistant will inform patient about the purpose, content, and points for attention of the trial; voluntariness; benefits and risks related to the study; principles of data protection and handling; and dissemination plans for the trial results. Then, the patient and/or immediate family may express their willingness to participate and sign the written informed consent of entering the trial. After enrollment in the cohort, all participates will provide consents for surgical treatments of the HCC, for entering a cohort randomly to receive a trial intervention, and for collecting and analyzing clinical, survival, and relevant medical data at each stage for the trial. Except for scientific research and communication with authorities in potential safety and regulatory issues, these consents have no plans for other purposes. Figure 1 and Table 1 present the study procedure and schedule for outcome assessment for the RCT.

**Table 1:**
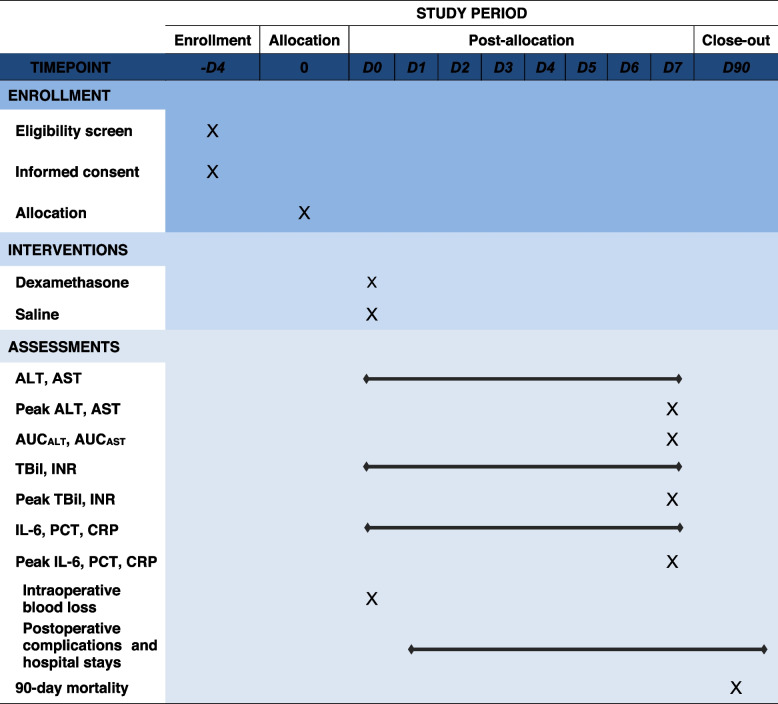
Schedule of enrollment, interventions, and assessments in the RCT

“×” indicates at which point of the RCT the respective assessments will take place

*RCT* randomized controlled trial, *ALT* alanine amine transferase, *AST* aspartate amine transferase, *AUC* area under the curve, *TBil* total bilirubin, *INR* international normalized ratio, *IL-6* interleukin-6, *PCT* procalcitonin, *CRP* C-reactive protein

### Inclusion criteria

All patients scheduled to undergo liver resection for HCC are considered as potential participants. Inclusion criteria are as follows: (1) IPM ischemic interval ≥ 25 min; (2) adequate functional reserves of important organ systems, New York Heart Association (NYHA) class I or II; pulmonary function is normal, mild, or moderate damage; chronic kidney disease (CKD) stage 1, 2, or 3; (3) normal liver function or well-reserved function before operation (Child–Pugh classes A or B with the score ≤ 7), (4) no other treatments for HCC before enrollment, such as portal vein embolization, transarterial chemoembolization, radiotherapy, targeted therapy, or immunotherapy.

### Exclusion criteria

Exclusion criteria are limited to patients: (1) aged < 18 years or > 80 years; (2) diseases receiving preceding systemic therapy with glucocorticoids, such as chronic kidney disease, inflammatory disease, or other immune system related diseases; (3) anesthesiologist judge that the subjects cannot use the dexamethasone, such as diagnosed with epilepsy or active ulcer; (4) intraoperative findings of extra-hepatic disease before intervention, need to undergo a synchronous resection for other organs except gallbladder; (5) intraoperative findings of additional lesions before intervention, need to combine with other procedures, such as ablation or bilo-enteric anastomosis; (6) unable to provide informed consent.

### Follow-up management and withdraw criteria

We will develop a rigorous follow-up plan to improve the follow-up rate and ensure data quality. Plans are as follows: (1) for subjects: excluding patients with inconsistent family opinions and unwillingness to be included in the screening process, strengthening subjects’ education in the informed consent process, and promoting communication with subjects of the time and content of review after the surgery and physical state after discharge; (2) for researchers: introducing dedicated personnel for follow-up management and dealing with the follow-up tables; (3) for information management: applying app software for follow-up management, developing a personalized follow-up management system. E-visit, a platform focused on patient clinical trials, supports patient original case upload, patient outcome report, follow-up management and doctor-patient interaction, various message reminders, and so on.

Participants will be allowed or required to withdraw from the RCT if the following are met: (1) they do not follow the treatment plan strictly (such as refusing continuous 7 days of collecting blood sample, receiving the targeted therapy or immunotherapy after surgery) while participating in the RCT, and (2) subjects are required to leave the RCT, such as participants cannot accept continuous 7 days of collecting blood sample or postoperative follow-up. For participants who withdraw from the RCT, we plan to record some routine postoperative testing indicators, including hepatocellular injury parameters (ALT, AST, and their peak value) and liver function indexes (TBil and international normalized ratio (INR)). Besides, postoperative complications, postoperative hospital stay, and 30- and 90-day mortalities are also recorded. However, these data will not be analyzed.

### Randomization and blinding

Once informed consent is provided, patients will be assigned randomly in a 1:1 ratio to the dexamethasone group or the control group on the day when they accept surgery. The computer-generated numbers are prepared by a research statistician and kept inside sealed envelopes by a research assistant who is not physically present in the operating room. Block size will not be disclosed to preserve allocation concealment.

When the patient enters the operating room, a research assistant will open the corresponding opaque envelope containing the patient allocation status and assigned medication. Then, the research assistant will refer the assigned medication to the anesthesiologist and remind them to inject the medication 10 min before IPM. The medication container, appearance, dosage, and technique of administration will be the same for two groups. Patients, surgeons, nursing team, anesthesiologists, and outcome assessors will be blinded to the study allocation status. If there is a situation such as a medical need or emergency which required unblinding, the principal investigator will be informed and make the final decision and report to the ethics committee.

### Intervention

Perioperative care and intraoperative technique for both study groups follow the routine practice of the surgical team. Patients in the dexamethasone group will receive intravenous administration of 10 mg dexamethasone (dexamethasone sodium phosphate injection, 1 ml:5 mg) 10 min before IPM. Patients in the control group will receive intravenous administration of 2 ml saline 10 min before IPM. All the patients will get routine postoperative rehabilitation management, and their relevant concomitant cares are homologous.

### Operative procedure

A low central venous pressure anesthesia (below 5 cmH_2_O) is required for hepatectomy. The types of incision include inverse L-shape under the right costal margin or “laparoscopic five hole.” Intraoperative ultrasound is routinely performed to identify, count, and characterize the nature and vascular proximity of the tumor. IPM is performed at the hepatoduodenal ligament using a rubber sling for both open and laparoscopic hepatectomy. The liver parenchyma is dissected by harmonic scalpel (Ethicon Endo-Surgery, Cincinnati, OH), and hemostasis is achieved with dipolar coagulation, clips, or suturing for either open or laparoscopic hepatectomy [10].

### Data collection

An outcome assessor will collect data from electronic medical record systems. All participants will be diagnosed with HCC at the outpatient clinic of West China Hospital of Sichuan University for the first time. Our planned recruitment period is from December 3, 2022, to April 1, 2023, with a postoperative follow-up of 3 months.

Demographic data:

- Age, year
- Gender, male/female
- Body mass index, kg/m^2^
- Comorbid conditions
- Liver disease background

Preoperative laboratory tests:

- Hepatitis B surface antigen positivity, *n* (%)
- Hepatitis B virus deoxyribonucleic acid ≥ 1000 IU/ml, *n* (%)
- Alpha-fetoprotein ≥ 400 ng/mL, *n* (%)
- TBil level, μmol/L
- ALT level, IU/L
- AST level, IU/L
- Albumin level, g/L
- INR
- Platelet count, 10^9^/L
- CRP level, mg/L
- IL-6 level, pg/ml
- Procalcitonin (PCT) level, ng/ml
- Child-Pugh grade A, *n* (%)
- Indocyanine green retention rate at 15 min ≤ 10%, *n* (%)
- Portal hypertension, *n* (%): defined as esophageal varices detected by endoscopy or a splenomegaly (major diameter > 12 cm) with a platelet count < 100,000/mm^3^ [20]

Intraoperative parameters:

- Tumor number, *n*
- Tumor size, cm
- Total operation time, min
- No. of the cycle of IPM, *n*
- Transection time, min
- Transection area, cm^2^: calculated the grids that the liver sections print on grid paper
- Total blood loss, ml: assessed by the extraction of blood in the suction apparatus subtract the amount of saline used for intraoperative irrigation plus the assessment amount of blood in the gauze roll (by weighting the soaked gauzes)
- No. of blood transfusion, *n* (%)
- Units of red blood cell transfusion, U
- Open/laparoscopic hepatectomy, *n*/*n*
- Type of resection: wedge resection, left lateral sectionectomy, right posterior sectionectomy, segmentectomy, bisegmentectomy, trisectionectomy, left hemi-hepatectomy, right hemi-hepatectomy, extended left/right hepatectomy
- Minor/major hepatectomy, *n*/*n*: defined as major when three or more segments are resected [21]

Postoperative laboratory parameters:

- Peak ALT, IU/L
- Peak AST, IU/L
- AUC_ALT_, U/(L×d)
- AUC_AST_, U/( L×d)
- Peak TBil, μmol/L
- Peak INR
- Peak CRP, mg/L
- Peak IL-6, pg/ml
- Peak PCT, ng/ml

Pathological data:

- Microvascular invasion, *n* (%)
- Tumor grade, *n* (%): G1, G2, G3-4
- Presence of fibrosis, *n* (%)
- Fibrosis stage, *n* (%): early (Ishak 1–2), intermediate (Ishak 3–4), advanced; cirrhosis (Ishak 5–6)

Postoperative follow-up data: surgeons and outcome assessor will jointly evaluate patients for postoperative complications and be blinded to the study allocation status of patients.

- Specific complications:
  * Liver failure: defined according to the “50-50 criteria” on postoperative day (POD) 5 [22]
  * Hemorrhage: defined as a postoperative hemoglobin level dropping more than 3 g/dL compared to the postoperative baseline level [23]
  * Bile leak: determined by an increase in bilirubin concentration in abdominal drainage of more than 3 times compared to that in serum on POD 3 [24]
  * Ascites: identified according to postoperative daily drainage fluid more than 10 mL/kg of body weight [25]
  * Pleural effusion: if symptomatic or requiring thoracocentesis or drainage
  * Incision site infection: if symptomatic or secretion culture positive
  * Other infections: diagnosed by responding clinical manifestation and etiological results
  * Other complications: classified and recorded according to the Clavien–Dindo grade [26]

- Adverse events (AE) and serious adverse events (SAE)
- No. of entering into intensive care unit, *n*
- Postoperative hospital stay, day
- 30-, 90-day mortality, *n* (%)

### Outcome measures

All blood samples will be collected on PODs 1, 2, 3, 4, 5, 6, and 7 following the same procedure for performing serial laboratory evaluation. All blood samples will be collected at 7 a.m. every day, which then will be sent by the outcome assessor to the Department of Experimental Medicine for examining by a fixed physician. Preoperative laboratory parameters are viewed as baseline of postoperative laboratory parameters (if the postoperative stay is not more than 7 days, outcome assessors will be responsible for the subjects for blood sample collection).

Primary outcomes are as follows: peaks value of ALT and AST and AUC of ALT and AST.

Indicators of hepatocellular injury are as follows: ALT and AST of postoperative 7-day both the two groups; then, we will calculate the mean for postoperative 7-day after surgery to depict the line charts with mean ± stand error.

Peak values of ALT and AST as well as AUC values of the postoperative 7-day course of ALT and AST (peak value is defined as the maximum value measured in 7 days, and AUC is calculated by the value of postoperative 7 days). The magnitude of the effect of glucocorticoids on relieving surgical pressure and alleviating ischemia-reperfusion injury are assessed by the AUC surrounded by continuous ALT and AST, which is the metrics for declaring superiority of the tested intervention.

We decided that peaks value of ALT and AST and AUC of ALT and AST were primary outcomes after 16 July 2023.

Secondary outcomes:

- Intraoperative blood loss
- Liver function: assessed by measuring serum level of TBil and INR for coagulation
- Inflammatory cytokines: assessed by IL-6, PCT, CRP, and their respective peaks
- Postoperative complications: according to the Clavien–Dindo grade, including major complications and minor complications
- Postoperative hospital stays
- Mortality: defined as in-hospital death or death within 30 or 90 days after surgery

### Safety and participant compensation

Although our intervention is convenient and less harmful, participants will be monitored for postoperative complications depicted in confirmed consent. We will care about patient and record each treatment until the AE is resolved. A participant who is found to be at risk to him/herself or others, or who has a SAE, will be referred to the relevant clinical services. Assessing the severity of the AE, SAE will be reported to the Biomedical Ethics Committee of West China Hospital, Sichuan University, within 24 h.

### Sample size

This trial is originally conceived by the statistical and clinical teams with a total of 270 patients (135 within each group). It is based on our previous RCT [10] and the other relative RCT in our center [27]. In our previous study, the standard deviation (*σ*) of the postoperative serum peak ALT value is 184.684, AST value is 141.1 between two groups, and the target difference (*δ*) is 58.882 IU/L. The sample size calculated using peak ALT value is larger than using peak AST value. Thus, we estimate a sample size of 135 patients in each group to achieve an 80% statistical power (Uβ = 0.84, 1-sided test) at the 5% significance level (Uα = 1.645, 1-sided test) as well as an estimated dropout rate of approximately 10%.

### Data management

Data will be recorded on case report form (CRF) in a timely, complete, and accurate manner by trained outcome assessors. CRF forms will be checked between outcome assessors. Then, they will input data into Excel software for the first time. Finally, another will input data for the second time; if the data is consistent, it can be submitted. Thus, electronic data will be stored and available to the outcome assessors only. Only they will have access to the data at the end of the RCT. Statistical analysis is conducted only after the trial. The Biomedical Ethics Committee of West China Hospital, Sichuan University, is responsible for monitoring the safety and process of the study.

### Statistical analysis

The trial is designed to test the superiority of dexamethasone to placebo with respect to the primary outcomes and secondary outcomes with the use of difference analysis. Superiority with respect to the primary outcomes are required before the secondary outcomes could be tested. All included participants will provide 80% power to detect superiority with respect to the primary outcomes. The AUC of ALT and AST will be tested for superiority, analyzed by Student’s *T* test or the Mann-Whitney *U* test.

Continuous variables will be presented as the mean ± standard deviation or median with its range based on normality testing and compared by an independent sample Student’s *T* test or the Mann-Whitney *U* test. Categorical variables will be presented as number (percentage) and compared using chi-square test or Fisher exact test. The modified intention-to-treat (ITT) principled analysis is applied for allocating patients into the group; moreover, when compliance is at a low level or the trial is no longer double-blinded, the as-treated (AT) analysis will be added to be supplements. Sensitivity analysis of missing data (loss to follow-up, death, withdrawal, etc.) will be conducted with the multiple-imputation approach. All tests for differences except primary outcomes are two-tailed and considered statistically significant if *P* values < 0.05. Bonferroni correction will be performed to correct for four primary outcomes statistically. We have changed the adjustment for multiple testing after 16 July 2023. All statistical analyses are performed by the SPSS version 20 and GraphPad Prism version 8.0.1.

As appropriate, the significance of postoperative categorical complications will be analyzed using chi-square test or Fisher exact test or will be tabulated and summarized using descriptive statistics.

Besides, according to the extent of resection and background of hepatic fibrosis after surgery, subgroup analysis will be performed to assess the protective effect of administration of dexamethasone before clamping the hilar pedicle on residual liver injury after undergoing liver resection with 25-min hepatic inflow occlusion.

### Oversight and monitoring

The surgeons and statisticians will be responsible for overseeing and monitoring the entire trial. Subject diagnosis, recruitment, and informed consent signing will be handled by assistant investigators. The progress, relevant events, and data quality of the trial will be evaluated by the Biomedical Ethics Review Committee of West China Hospital, Sichuan University. There will be no interim analysis. If individual participants report SAEs during the course of the trial, the principal investigator will make a decision to terminate the trial. The study team will report monthly of the data to committee for monitoring the progress and quality of the study.

### Patient and public involvement

All the research question and outcome measures are according the objective test results and facts. Patients and the public will not be involved in the study design, recruitment, implementation, reporting, or assessing. However, the study results will be disseminated to the public through academic papers and conferences.

## Discussion

IPM is a crucial technique applied in liver resection; it has been considered the gold standard for controlling bleeding during liver transection [28]. Some studies manifest that liver resection without hepatic pedicle clamping is safe and IPM does not reduce blood loss [29, 30], but when we plan to perform a major hepatectomy or laparoscopic resection, should it be with or without IPM? The answer is ambiguous. We are confronting two existing issues: [1] HCC patients usually have underlying liver diseases such as cirrhosis or portal hypertension in our country, and [2] laparoscopic hepatectomy tends to be advanced and prevailing. Therefore, IPM remains a priority for controlling bleeding to maintain a clear operative view and reduce the rate of open conversion. In this regard, we designed a RCT of prolonging the IPM time to 25 min in hepatectomy for HCC, concluding that 25-min IPM group had significantly higher speed for parenchyma transection and less blood loss under the premise that residual liver injury was not significantly aggravated. These benefits were more pronounced in laparoscopic hepatectomy [10]. In addition, we observe that perioperative glucocorticoid administration has been studied intensively, and many studies have shown that perioperative glucocorticoid administration alleviates hepatic injury, resists inflammation, and decreases morbidity and mortality rate for patients undergoing liver resection [13–18, 31]. However, these reports included, but were not restricted to, inconsistent glucocorticoid preparation, timing of administration, and dosage. In a word, this trial protocol is based on our previous study to explore further the effect of glucocorticoid administration on attenuating the surgical stress response in order to follow securely 25-min hepatic inflow occlusion.

Of course, we also knowledge that preoperative steroids administration may arise some negative effects, such as inhibition of liver regeneration, gastrointestinal bleeding, and infection. However, Glanemann et al. have demonstrated that preoperative steroids administration has no apparent effects on hepatic regeneration [32]. Furthermore, liver regeneration has a close relationship with IL-6 that the overproduction of IL-6 inhibits liver regeneration [33]. As for gastrointestinal bleeding, dexamethasone administration should be cautious in patients after hepatectomy [34]. We will routinely use proton pump inhibitors. Administration of glucocorticoids may cause postoperative infections due to elevation of blood glucose. However, a recent RCT demonstrates that administration of preoperative single-dose methylprednisolone in patients undergoing a subsequent major liver resection lowers the risk of postoperative complications and surgical site infections as well as results in a shorter length of hospital stay [35]. Therefore, the trial protocol is reasonable and the results of the trial may be clinically significant.

### Trial status

The trial has been reviewed and approved by the Biomedical Ethics Review Committee of West China Hospital, Sichuan University, on 28 November 2022 (ethics reference: 2022(1757)). The protocol version is V1.0 on 4 November 2022. This trial began to recruit patients on 3 December 2022. Recruitment was complete on 1 April 2023. Follow-up of all patients was complete on 1 July 2023. The data were unblinded on 16 July 2023. The final version of this protocol has been submitted for publication on 19 November 2023.

### Supplementary Information

**Additional file 1.** Consent informed form

## Data Availability

At present, the datasets are not readily available. Data will be available from the corresponding author by reasonable request after study completion. A relevant report with study results will be submitted for publication in an appropriate journal, approximately 3 months after finishing the data collection.

## Abbreviations

RCT: Randomized controlled trial
IPM: Intermittent Pringle’s maneuver
HCC: Hepatocellular carcinoma
IRI: Ischemia-reperfusion injury
AUC: Area under the curve
TBil: Total bilirubin
PT: Prothrombin time
CI: Confidence interval
IL-6: Interleukin-6
IL-10: Interleukin-10
TNF-α: Tumor necrosis factor-α
CRP: C reactive protein
AST: Aspartate transferase
ALT: Alanine transferase
NYHA: New York Heart Association
CKD: Chronic kidney disease
INR: International normalized ratio
PCT: Procalcitonin
POD: Postoperative day
AE: Adverse event
SAE: Serious adverse event
CRF: Case report form
ITT: Intention-to-treat
AT: As-treated
