# Supplementary material for: Preoperative dexamethasone administration in hepatectomy of 25-min intermittent Pringle’s maneuver for hepatocellular carcinoma: protocol for a randomized controlled trial
Source: Trials. 2023 Nov 30;24:774. doi: 10.1186/s13063-023-07820-0 (PMC10691107; doi:10.1186/s13063-023-07820-0)
Supplement: Supplementary file 1 — Additional file 1. Consent informed form [file 13063_2023_7820_MOESM1_ESM.pdf]

West China Hospital, Sichuan University

Participant Informed Consent

We invite you to participate in the research of "a RCT study of the effect on preoperative outcomes of dexamethasone applied to patients undergoing hepatectomy with 25-minute intermittent Pringle's maneuver ", approved by West China Hospital of Sichuan University. It is estimated that 270 subjects will participate voluntarily. This study has been reviewed and approved by the Biomedical Ethics Committee of West China Hospital of Sichuan University.

Name: **Gender:** **Age:** **Inpatient ID:**

1. Why should we carry out this study?

Hepatectomy is a radical treatment for hepatocellular carcinoma (HCC). In the 20th century, the mortality rate after operation reached more than 20%. With the development of modern surgical instruments, surgical technology and perioperative management, hepatectomy has been greatly promoted and improved, and its safety has been greatly improved, and the mortality rate has dropped to about 5%. Due to the improvement of its safety, the application of hepatectomy has been expanded, such as intrahepatic bile duct stones, liver abscess, hepatic hemangioma, liver focal hyperplasia and liver hydatid. The complications after hepatectomy mainly include liver failure, bile leakage, hemorrhage, ascites and infection. Among them, the factors influencing postoperative complications include operation time, tumor size, intraoperative bleeding, general state of patients before surgery, etc. At present, blocking the inflow of liver is the most commonly used methods to reduce intraoperative bleeding during hepatectomy. The most commonly used methods to block the blood inflow of liver is intermittent Pringle's maneuver (IPM). Ischemia-reperfusion injury (IRI) exists when the blood inflow of liver is blocked. Normal liver can tolerate ischemia for 30 minutes. Therefore, compared with the routine 15-minute IPM, we concluded in the previous study that patients undergoing laparoscopic or open hepatectomy with 25-min IPM does not suffer more severe liver injury, and prolonging the ischemic time significantly reduces intraoperative blood loss and improves the speed of hepatectomy in the process of hepatectomy. There is evidence that administration of glucocorticoids can significantly reduce IRI. Therefore, this study is mainly on the basis of 25-min IPM, and further explore to give dexamethasone 10mg intravenous injection 10 minutes before blocking the blood inflow of liver, and comparing liver function, intraoperative condition and the incidence of postoperative complications with the control group.

2. What do you need to do if you take part in the study?

You may be randomly assigned to experimental group (with dexamethasone) or control group (without dexamethasone). You need to sign the relevant informed consent form preoperatively, and the following items should be checked

preoperatively: blood routine, blood biochemistry, coagulation function, tumor marker and cardiopulmonary and renal function assessment.

3. What are the treatment options available?

Surgical resection, radiofrequency ablation, transcatheter arterial chemoembolization, liver transplantation, radiation therapy or systemic therapy. During hepatectomy, blood flow occlusion will be performed in addition to Pringle's maneuver (used in this experiment), as well as hemihepatic inflow occlusion and selective hepatic inflow occlusion, ect.

4. Who should not be included in the study?

(1) age < 18 years old, (2) diseases receiving preceding systemic therapy with glucocorticoids, such as chronic kidney disease, inflammatory disease or other immune system related diseases, (3) intraoperative findings of extra-hepatic disease, need to undergo a synchronous resection for other organs except gallbladder, (4) intraoperative findings of additional lesions, need to combine with other procedures, such as ablation or bilo-enteric anastomosis.

5. What are the common postoperative complications and therapeutic measures?

Postoperative complications include: 1. postoperative bleeding: less bleeding is treated with hemostatic agents, blood transfusion and other symptomatic treatments, and more bleeding requires intervention or laparotomy to explore for hemostasis. 2. biliary leakage: according to the situation of biliary leakage, less biliary leakage guarantees adequate drainage, more biliary leakage requires intervention or ERCP, exploratory laparotomy. 3. pulmonary infection: long-term bed rest after surgery is easy to cause pulmonary infection, encouraging expectoration, anti-infection and respiratory support treatment. 4. abdominal infection: puncture and drainage, anti-infection treatment. 5. incision infection: patency drainage, dressing exchange frequently. 6. incision pain: relieving pain by injection, analgesic pumps. 7. ileus: encouraging patients to get out of bed and promote gastrointestinal motility, if necessary, chinese acupuncture, gastrointestinal decompression, enema, intestinal adhesiolysis and other treatments.

6. What are the possible benefits of participating in the study?

Participating in this study may be conducive to prove to be effective in avoiding more severe postoperative liver injury when using dexamethasone when prolonging the time of IPM to 25min, while our findings will be applied more effectively to other patients with similar conditions to you.

7. Are there any costs?

The only intervention in this study is whether to use dexamethasone or not, and the current price of dexamethasone injection in our hospital is 0.3 RMB/ branch (5mg), so patients in the experimental group will pay additional 0.6 RMB compared with the control group.

8. Is personal information confidential?

Your research materials will be maintained at West China Hospital, Sichuan University, and your medical records will be available to the investigator, the research authority, and the ethics review board. Any public reporting of the findings of this study will not disclose your personal identity. We will make every effort to protect the privacy and personal information of your personal medical data to the extent allowed by law.

9. Do I have to take part in the study?

Participating in this study is completely voluntary and you may refuse to participate in the study or withdraw from it at any time during any phase of the trial without discrimination and reprisal whose medical treatment is unaffected from equity. If you decide to withdraw from this study, please contact with us for proper diagnosis and treatment of the disease.

Subjects' statement: I have read the above introduction to this study and my researchers have fully explained to me the purpose of this study, the operational procedure and the possible risks and potential benefits of participating in this study and answered all my relevant questions.

Consent☐ or Refusal☐ studies other than this study utilize my research materials and biological specimens.

Signature of participant: Date：

Signature of participant's family: Date：

Phone number:

Doctor's statement: I have given relevant details of this study to the above volunteers who participated in this study and provided him / her with an original copy of a signed informed consent form. I confirm that the circumstances of this study have been explained to the subjects in detail, in particular, the ethical principles and

requirements such as possible risks and benefits, free and compensation, damages and compensation, voluntary and confidentiality.

Signature of doctor: Date：

Phone number:

Biomedical ethics review board of West China Hospital, Sichuan University

Phone number：028-85422654，028-85423237
